# Progressive disseminated histoplasmosis with concomitant disseminated nontuberculous mycobacterial infection in a patient with AIDS from a nonendemic region (California)

**DOI:** 10.1186/s12890-019-0808-8

**Published:** 2019-02-21

**Authors:** Julian Choi, Kia Nikoomanesh, Jusleen Uppal, Sharon Wang

**Affiliations:** 1 Department of Pulmonary and Critical Care Medicine, Arrowhead Regional Medical Center, Colton, USA; 2 Department of Internal Medicine, Arrowhead Regional Medical Center, Colton, USA; 3 Department of Infectious Disease, Arrowhead Regional Medical Center, Colton, USA

**Keywords:** Histoplasmosis, AIDS, Nontuberculous mycobacterial infection, Nonendemic, Opportunistic infections, Reactivation, California, Co-infection

## Abstract

**Background:**

Opportunistic infections, while well studied in the AIDS population, continue to have variable and surprising presentations. Here, we present a case of disseminated histoplasmosis with disseminated nontuberculous mycobacterial infection in a 50 year old man with long standing AIDS living in a non-endemic area.

**Case presentation:**

Patient presented with a constellation of symptoms, and imaging of the chest showed a pulmonary mass with cavitation, multiple nodules, and ground glass opacities. Further investigations revealed granulomatous lung nodules and fungemia consistent with *Histoplasma capsulatum*, and coinfection with disseminated nontuberculous mycobateria in a nonendemic area.

**Conclusions:**

Immunocompromised patients risk co-inhabitation by multiple infectious organisms. Some of these organisms may preside in the host for years prior to reactivation. Clinicians in non endemic areas should therefore be careful to not overlook specific organisms based on a lack of a recent travel history. Physicians in nonendemic areas should become more familiar with the clinical findings and diagnostic approach of infectious such as Histoplasmosis, to ensure earlier recognition and treatment in immunocompromised individuals.

## Background

*Histoplasma capsulatum* is an endemic, dimorphic fungus commonly found in the midwest and southern United States (US), and most prevalent along the Ohio and Mississippi river valleys. It is the most common cause for hospitalization of the endemic mycoses [1]. Patients with acquired immune deficiency syndrome (AIDS) have weakened cellular immunity and increased risk for histoplasmosis especially in areas of endemic foci. However, in nonendemic areas such as California (where our patient resides), it is thought to be due to reactivation of latent infection from previous exposure rather than an acute infection [2–5]. Clinical presentation depends on the immune status of the host and extent of exposed inoculum. Healthy individuals are typically asymptomatic or have self-limiting infections, while many with AIDS present with progressive disseminated histoplasmosis with pulmonary involvement [1, 4, 6]. Herein we present a patient with AIDS coinfected with disseminated nontuberculous mycobacterial infection and progressive disseminated histoplasmosis in southern California.

## Case presentation

A 50-year-old homeless Caucasian man with history of AIDS presented for generalized weakness and productive cough with clear-yellow sputum without hemoptysis for 1 month. He also endorsed fevers, chills and rigors for 1 week and a 15 pound unintentional weight loss in 1 month. AIDS was diagnosed over 20 years ago and has been noncompliant with various combinations antiretroviral therapy (cART) regimens including emtricitabine/tenofovir, abacavir/lamivudine, darunavir, and ritonavir. Patient was lost to follow-up for 2 years until he was recently incarcerated and released from jail. Patient was born in Ohio but moved to California at 2 years of age, and had remote military service in Georgia in his early 20’s. Otherwise the patient never left California thereafter. He has never explored caves or been in contact with birds, bats or its excrements.

Patient’s initial temperature was 38.5**°** Celsius and he was also tachycardic. Physical exam revealed a disheveled, cachectic male with temporal muscle wasting, no respiratory distress on room air, and was otherwise unremarkable.

**Fig. 1:**
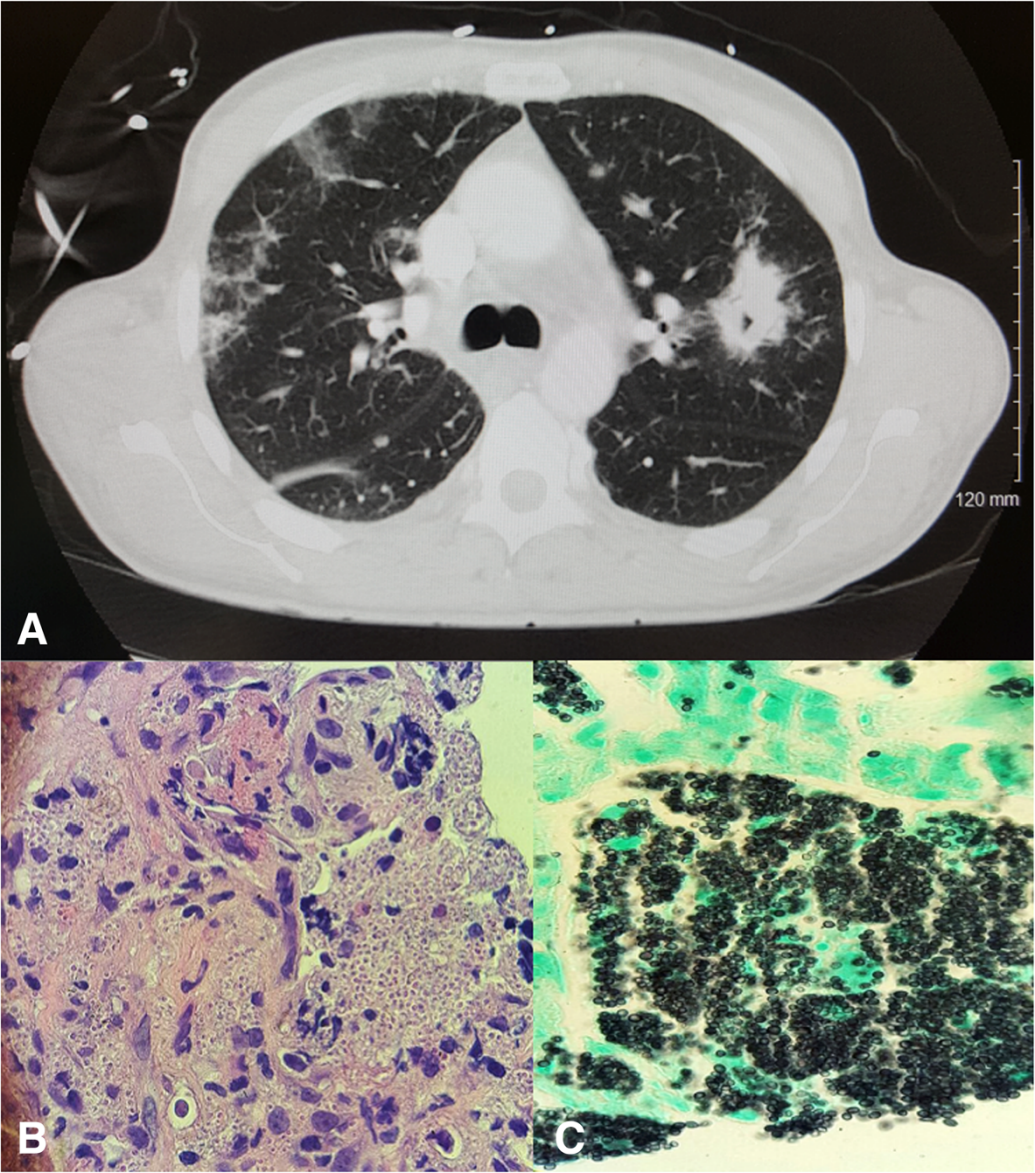
**a** CT chest showing left lung cavitary mass, nodular and ground-glass opacities, and mediastinal lymphadenopathy. **b** Hematoxylin and eosin (H&E) stain showing granuloma formation and dimorphic yeast. **c** Gomori Methenamine silver (GMS) stain showing numerous dimorphic oval-shaped budding yeast consistent with *Histoplasma capsulatum*

Laboratories revealed a white blood cell count of 3.7 TH/uL, absolute lymphocyte count of 185, absolute CD4 count of 20 cells/uL, and HIV viral load of 181,000 copies/mL. Comprehensive metabolic panel was within normal ranges except for a low albumin (2.8 g/dL). Lactate dehydrogenase (277 u/L), ferritin (1343 ng/mL), erythrocyte sedimentation rate (111 mm/hr), and C-reactive protein (9.58 mg/dL) were elevated. Computed tomography (CT) of the chest with contrast revealed bilateral nodular opacities, the largest measured (3.6 × 2.2 cm), a left upper lobe mass with cavitation, right basilar (2.0 × 1.5 cm) nodule, mediastinal lymphadenopathy, ground-glass changes, and enlarged periaortic lymph nodes (Fig. 1a). CT abdomen and pelvis revealed retroperitoneal lymphadenopathy and was otherwise unremarkable.

The initial workup included a lumbar puncture which was unremarkable. Multiple sputum acid fast bacilli (AFB) smears were negative. Interestingly, serum and urine were positive for rare AFBs using fluorochrome staining but nucleic acid amplification testing (NAAT) for *Mycobacterium tuberculosis* complex were negative, raising suspicion for disseminated nontuberculous mycobacterial (NTM) infection. Patient was started on treatment for presumed disseminated NTM infection with daily regimen of azithromycin/rifabutin/ethambutol with resolution of fever. However, patient still endorsed a cough. He subsequently underwent image guided fine needle aspiration of peripheral lung nodules. Pathology (Fig. 1b & c) revealed granulomatous inflammation with abundant *Histoplasma capsulatum* organisms by Grocott-Gomori’s methenamine silver stain (GMS) staining. There were no AFB positive organisms seen. Of note, the diagnosis was further supported by positive urine *Histoplasma* antigen (> 19 ng/ml) and serum *Histoplasma* antigen above the limit of quantification. Histoplasma antibody with mycelial antigen was < 1:8. Blood and final bronchoscopy cultures eventually grew *H. capsulatum* weeks later.

Once diagnosis of *H. capsulatum* was made, in addition to NTM treatment regimen, the patient was also started on intravenous amphotericin B lipid suspension at 5 mg/kg for 2 weeks and subsequently discharged with itraconazole. Patient was also started on cART (emtricitabine/tenofovir/dolutegravir) prior to discharge. With treatment, all constitutional symptoms and laboratory abnormalities improved. He was subsequently discharged home with close outpatient follow-up.

## Conclusion

It is well known that *Histoplasma capsulatum* is endemic to the Ohio and Mississippi river valley regions of the US. According to articles from the Centers for Disease Control and Prevention (CDC), between the years 1938 and 2013, there were 2850 reported cases of histoplasmosis spread out across 26 states of the US [7]. Of those cases, none were found in California. This seems to be similar for the years 2011–2014, with 3409 reported cases in 12 states, none being in the west coast [8]. Although the CDC does not list *H. capsulatum* to be a reportable fungal disease in California, one could presume that identifying histoplasmosis in California (and other nonendemic states) is rare, or, underdiagnosed and underreported.

In the era of cART therapy HIV infected individuals have the opportunity to live longer and travel around the globe, potentially increasing the opportunity of making a diagnosis of histoplasmosis in nonendemic areas [9]. Histoplasmosis is a common opportunistic infection in endemic regions for individuals with AIDS. They are at higher risk for histoplasmosis with up to 25% occurrence in select cities compared to 1% in nonendemic areas [6, 10].

Clinical presentation of histoplasmosis depends on the volume of exposure, host immune status, and overall health. Histoplasmosis can be the first manifestation of AIDS in up to 50–75% of patients with disseminated infection with mortality rates as high as 39–58% depending on endemicity [9]. Traditionally, progressive disseminated histoplasmosis (PDH) presents with myriad of non-specific symptoms and examination findings: fevers, night sweats, weight loss, cough, dyspnea, fatigue, hepatosplenomegaly, lymphadenopathy, and less frequently, skin, gastrointestinal, and central nervous system manifestations [1, 4, 6]. PDH is usually seen in AIDS patients with absolute CD4 counts of less than 150–200 cells/uL [9, 11]. Other associated laboratory values (although non-specific) include: pancytopenia, elevated ferritin, lactate dehydrogenase, and abnormal liver function tests [1–3, 12].

Histoplasmosis diagnosed in nonendemic regions, as seen with this particular case, was likely from reactivation of latent infection in those without recent exposure [2, 3, 5, 10, 13]. Our patient likely was exposed in Ohio during his first 2 years of life, or in Georgia prior to traveling to southern California. His detailed travel history accounted for no other visits to endemic areas for over 20 years. He was also diagnosed with HIV for 20 years and was compliant in the past with antiretroviral therapy until about 2 years from presentation.

There is high variability between time of travel to endemic areas and manifestations of clinical symptoms. One survey in Europe described symptoms occurring between 2 months to 5 years after travel to endemic area [14]. They also describe individuals in the United Kingdom who served in World War II in endemic areas who had latency periods more than 50 years before reactivation. It is prudent that a detailed travel history be obtained to elicit past exposures from endemic areas to not delay recognition and treatment for histoplasmosis. During that time he likely had declining cellular immunity and endogenous reinfection from weakened host defenses. Furthermore, there are reports of certain biologic medications being associated with reactivation occurring in nonendemic regions that may be related to decreased immunity [5].

Clinicians in nonendemic areas may not consider histoplasmosis as a diagnosis especially if patients have co-infections that could potentially account for clinical and laboratory irregularities. However, multiple concurrent infections are very common in patients with AIDS. [6, 13, 15, 16]. Fredericks et. al. reviewed 46 patients from two San Francisco hospitals with disseminated histoplasmosis and AIDS. 54% had coinfections and only 1 of the cases listed histoplasmosis as part of their original differential diagnosis [3]. Kitkungvan et al. performed an observational study in a Thailand hospital showing 26% of their HIV infected individuals presenting with fever of unknown origin to have co-infections with 2 or more pathogens [17]. The principle of “Occam’s razor” is that the simplest solution is often correct. However, in patients with AIDS, this can misguide investigators in making erroneous assumptions. Instead, it may better serve clinicians caring for these immunocompromised individuals to apply the concept of “Hickam’s dictum.” It is a paradigm in which a patient’s manifestations are better explained not by a single unifying diagnosis, but multiple diseases processes occurring at once. In the era of cART and increased global travel, endemicity of a particular infectious disease may no longer be localized (as in our case) to a specific region. Physicians in nonendemic areas should become more familiar with the clinical findings and diagnostic approach of histoplasmosis, to ensure earlier recognition and treatment for this vulnerable group of individuals.

## Abbreviations

AFB: Acid Fast Bacilli
AIDS: Acquired immune deficiency syndrome
cART: Combinations antiretroviral therapy
CD4: Cluster of differentiation 4
CDC: Center for Disease Control and Prevention
Cells/uL: Cells per microliter
cm: Centimeter
g/dl: Grams per deciliter
mg/dl: Milligrams per deciliter
Mg/kg: Milligram per kilogram
mm/hr.: Millimeters per hour
NAAT: Nucleic acid amplification test
ng/ml: Nanogram per milliliter
NTM: Nontuberculous mycobacterium
PDH: Progressive disseminated Histoplasmosis
u/L: Units per liter
